# Improvement of social functioning in patients with first-episode schizophrenia using blonanserin treatment: a prospective, multi-centre, single-arm clinical trial

**DOI:** 10.3389/fpsyt.2024.1345978

**Published:** 2024-03-20

**Authors:** Tianqi Gao, Hong Deng, Jianhua Sheng, Bin Wu, Zhening Liu, Fude Yang, Lina Wang, Shaohua Hu, Xijin Wang, Haiyun Li, Chengcheng Pu, Xin Yu

**Affiliations:** ^1^ Peking University Sixth Hospital, Peking University Institute of Mental Health, National Health Commission (NHC) Key Laboratory of Mental Health (Peking University), National Clinical Research Center for Mental Disorders (Peking University Sixth Hospital), Beijing, China; ^2^ Mental Health Center, West China Hospital of Sichuan University, Chengdu, China; ^3^ Department of Psychiatry, Shanghai Mental Health Center, Shanghai Jiao Tong University School of Medicine, Shanghai, China; ^4^ Department of Psychiatry, Xi’an Mental Health Center, Xi’an, China; ^5^ Mental Health Institute of the Second Xiangya Hospital, Key Laboratory of Psychiatry and Mental Health of Hunan Province, Central South University, The State Key Laboratory of Medical Genetics, Central South University, Changsha, China; ^6^ Psychiatry Research Center, Beijing Huilongguan Hospital, Beijing, China; ^7^ Department of Psychiatry, Tianjin Anding Hospital, Tianjin, China; ^8^ Department of Psychiatry, The First Affiliated Hospital, Zhejiang University School of Medicine, Hangzhou, China; ^9^ Department of Psychiatry, The First Psychiatric Hospital of Harbin, Harbin, China; ^10^ Medical Affairs, Sumitomo Pharma (Suzhou) Co., Ltd., Shanghai, China

**Keywords:** blonanserin, first-episode schizophrenia, social functioning, symtomatic and neurocognitive improvement, clinical trial

## Abstract

**Objectives:**

This clinical trial primarily aimed to investigate the effects of blonanserin on social functioning in patients with first-episode schizophrenia.

**Methods:**

In this prospective, multi-centre, single-arm clinical trial study, blonanserin (flexible oral dose ranging from 8mg to 24mg per day) was given 26 weeks. Outcome measures included the Personal and Social Performance (PSP) scale for evaluating social functioning, the Measurement and Treatment Research to Improve Cognition in Schizophrenia Consensus Cognitive Battery (MCCB) for measuring neurocognitive performance, and the Positive and Negative Syndrome Scale (PANSS) for assessing symptom severity. The primary endpoint was social function improvement evaluated by PSP scale at the end of blonanserin treatment. And the secondary endpoint was to validate the efficacy and neurocognitive effects of blonanserin. Adverse drug reactions (ADRs) were also recorded and analysed.

**Results:**

A total of 96 patients with first-episode schizophrenia were recruited and proceeded to analysis. Fifty-one participants (53.1%) completed the PSP scale measurements at baseline and week 26. Following 26 weeks of blonanserin treatment, all outcome measurements demonstrated significant improvement during the follow-up period. Notably, PSP scores exhibited a continuous increase up to 68.1% ± 103.7% at the end of the treatment (46.6 ± 14.6 at baseline, 69.4 ± 17.4 at week 26, p<0.001), indicating positive effects on social functioning that were already noticeable by week 8.

**Conclusion:**

Blonanserin treatment exhibited favourable effects on social functioning in individuals with first-episode schizophrenia. The results suggest that blonanserin was effective treatment options for patients with schizophrenia encountering functional impairments.

## 1 Introduction

Schizophrenia is a severe and chronic mental disorder characterized by disturbances in social functioning and perception. The global population with onset typically occurring in late adolescence or early adulthood with prevalence estimates ranging from 0.5 to 1% (1–3). Patients often experience significant impairments in social functioning, which leads to a diminished quality of life (4, 5). One of the key challenges in treating schizophrenia is the successful treatment of positive symptoms has not been demonstrated to dramatically improve functional outcomes, such as social and employment relationships (6). Therefore, improving social functioning has become one of the important targets for intervention.

While antipsychotic medications have been the cornerstone of treatment for schizophrenia, their effectiveness in addressing the broader of social and neurocognitive deficits associated with the disorder remains limited. Neurocognitive symptoms are not particularly amenable to treatment with the currently marketed antipsychotics, evidence demonstrates an agent that can improve cognitive symptoms and thereby improve functional outcome are still limited to date. Therefore, there is a growing interest in identifying new pharmacological interventions that can target these specific domains and enhance the overall functional outcomes for patients with schizophrenia.

Blonanserin, a novel atypical antipsychotic agent, has emerged as a potential therapeutic option for schizophrenia. By intricately modulating both dopamine D2/3 and serotonin 5-HT2A receptor subtypes within the brain, blonanserin targets key elements that are widely recognized to play a crucial role in the pathophysiology of the disorder (7). This dual modulation of neurotransmitter systems signifies blonanserin’s potential to address the intricate neurochemical imbalances that contribute to the diverse symptoms and manifestations of schizophrenia (7–9). Previous studies have suggested that blonanserin have a favourable profile in terms of reducing both positive and negative symptoms of schizophrenia, and associated with relatively minor side effects (7, 10, 11).

Neurocognitive impairment in schizophrenia is different from positive and negative symptoms (4). Despite psychotic symptom improvement, long-term studies reveal enduring neurocognitive impairment linked to social dysfunctions (5). Thus, enhancing social and neurocognitive functions remains a key therapeutic aim. However, the evidence of blonanserin’s impact on these aspects in schizophrenia was limited, with its availability restricted to the markets of Japan, South Korea, and China etc. Their effects have only been investigated in a few clinical trials with sample sizes less than 40 patients (10–12). Moreover, there is a paucity of research focusing on the group of first-episode schizophrenia to investigate the effects of blonanserin on social functioning. To better understand the impact of blonanserin on these crucial aspects of the illness, we aimed to explore the effects of blonanserin on social functioning and its efficacy in patients with first-episode schizophrenia in a prospective, multi-centre, single-arm clinical trial.

## 2 Methods

### 2.1 Study design

This study was a prospective, multi-centre, single-arm clinical trial, ethically considering the use of placebo was not recommended for patients with first-episode schizophrenia (13), as previously described in detail (14). From January 2019 to September 2022, patients were recruited from 9 hospitals in China. The 26-week follow-up was completed in November 2022. The baseline and washout period assessment included the collection of relevant variables such as demographic data, medical history, medication history, living history (including living habits and social psychological habits), smoking history, family history, suicide history, vital signs, laboratory tests, pregnancy tests, electrocardiogram (ECG), Social Performance Scale (PSP), Measurement and Treatment Research to Improve Cognition in Schizophrenia Consensus Cognitive Battery (MCCB), and Positive and Negative Syndrome Scale (PANSS).

This study received approval from the ethics committee of the leading site, Peking University Sixth Hospital (No. 2018-18), and it was registered on ClinicalTrials.gov (No.: NCT03784222). Prior to enrolment, all participants provided written informed consent. The study adheres to the principles of the Declaration of Helsinki and complies with all existing guidelines for clinical research.

### 2.2 Participants

Patients were eligible for the study if they met the following criteria: (1) schizophrenia diagnosed according to Diagnostic and Statistical Manual of Mental Disorders-5 (DSM-5) or International Classification of disease-10 (ICD-10); (2) PANSS total score ≥70; (3) age of 18-45 years; (4) first-episode patients with disease course less than 5 years; (5) ≥9 years of education; (6) no prior systematic antipsychotic treatment, single continuous treatment less than 6 weeks, or total treatment duration less than 6 months; (7) capable of reading and understanding Chinese characters; (8) provide written informed consent. Patients excluded from the study were previously described in detail (14).

### 2.3 Intervention and assessment

Before starting blonanserin (Sumitomo Pharma (Suzhou) Co., Ltd., Suzhou, China) treatment, patients would undergo a washout period of 0-7 days. If patients haven’t received any previous antipsychotic treatment, they may be exempted from the washout period. The initial dosage was 4mg per administration, taken orally twice a day after meals. The dosage can be gradually increased thereafter. The maintenance dosage was 8-24 mg per day, divided into two administrations after meals. The dosage may be adjusted based on the patient’s age and symptoms, but the daily dosage should not exceed 24mg. Assessments were conducted on the following weeks: 0, 2, 4, 8, 12, 16, 21 and 26 weeks. Throughout this period, assessment data, adverse effects (AEs), and concomitant medications were consistently documented. Moreover, some drugs were prohibited during the study period, as previously described in detail (14).

### 2.4 Outcome measures

In the present study, 3 outcome measures were assessed: PSP, MCCB and PANSS. The PSP served as the primary outcome measure. Following the intervention, changes in the outcome measures were calculated from baseline to the respective visits (e.g., week 2, week 8, week 12, and week 26, etc., see **Supplementary Table 1**). The MCCB and PANSS served as the secondary outcome measures.

The PSP was developed to assess the severity of the patient’s performance across 4 domains: socially useful activities, personal and social relationships, self-care, and disturbing/aggressive behaviour (15). The scores of individual subscales were summed, resulting in a maximum possible score of 100, with higher scores indicating better personal and social functioning. Early response of PSP is defined as PSP total score increase ≥ 7.62 at week 8 from baseline, as an increase of 7.62 was reported the minimum clinically important difference of PSP in patients with acute schizophrenia (16).

The MCCB is widely utilized for evaluating neurocognitive deficits in schizophrenia. It encompasses 7 cognitive domains: attention, information processing speed, verbal learning and memory, visual learning and memory, working memory, reasoning and problem-solving, and social cognition (17). The Global Deficit Score (GDS) method was employed to classify overall impairment status on the MCCB battery. It can be analysed as a continuous variable indicating number and severity of neurobehavioral deficits across the entire test battery, or as a cut-off of ≥0.50 that can be used to classify overall impairment. In other words, the higher GDS is linked to more severe neurocognitive impairment (17, 18).

PANSS was employed to assess positive, negative, and general psychopathology symptoms in individuals with schizophrenia (19). Participants were rated the severity of symptoms using a set of 30 items scored from 1 to 7. Higher scores indicate greater severity of psychotic symptoms. The minimal response was defined as a reduction in the PANSS total score of ≥ 20%, while a PANSS response was defined as a reduction of ≥ 50% in the PANSS total score. A reduction rate was calculated using the following formula: reduction rate = (baseline PANSS total score - current PANSS total score)/(baseline PANSS total score - 30) × 100%.

### 2.5 Safety and surveillance

Safety indicators include: (1) incidence of adverse drug reactions (ADRs); (2) changes in body weight, body mass index (BMI), blood glucose, and blood lipid levels compared to baseline; (3) changes in serum prolactin concentration; 4. scores on the Arizona Sexual Experience Scale (ASEX); 5. routine urine analysis, liver and kidney function tests, vital signs, and 12-lead electrocardiogram; 6. scores on the Simpson-Angus Scale (SAS)/Barnes Akathisia Rating Scale (BARS)/Abnormal Involuntary Movement Score (AIMS). When assessing the total ADRs, the count was based on the most severe symptom experienced by patients.

### 2.6 Analysis sets

The full analysis set (FAS) encompasses data from all patients included in the study who have used blonanserin at least once. The primary endpoint was assessed using the data from subjects who completed the PSP scale measurements at baseline and week 26, while secondary efficacy indicators were analysed using the actual data within the FAS.

The safety set (SS) comprises the dataset containing the recorded post-treatment safety indicators of patients who have received at least 1 treatment. The number of patients in the SS served as the denominator for calculating the incidence of adverse reactions.

Exploratory analyses were also conducted. The selection of participants was based on the analysis requirements such as age, gender, duration of disease, baseline PSP, MCCB, PANSS, and mean daily dose of blonanserin in the first 2 weeks.

### 2.7 Statistical analysis

Differences before and after treatment were estimated using either mean and standard deviation (SD) or median and range for continuous variables. The p values were obtained from pairwise t-tests or Wilcoxon signed rank tests, depending on the normality of the distribution. For categorical variables, the number and percentage were used, and the chi-square test (x^2^ test) was applied. Changes in outcome measures at various time points in the treatment group were analysed using repeated measures analysis of variance. To assess the associations of relevant factors with the early response of outcome measures, multivariate logistic regression models were used.

All analyses were conducted using R software (version 4.2.1) or Stata/SE14.2 (Stata, College Station, TX). All statistical tests were two-sided and used a significance level of p < 0.05, unless otherwise indicated.

## 3 Results

The baseline characteristics of study participants are presented in **Table 1**. Among the 96 patients included in the study (43 men and 53 women), the mean age at recruitment was 27.5 ± 7.0 years. During the 26-week follow-up period, all patients received a daily average treatment dose of blonanserin at 14.0 ± 4.8 mg.

**Table 1:** Baseline characteristics of participants (FAS, n=96).

Variables	Mean ± SD or N (%)
Age (Years)	27.5 ± 7.0
Gender	
Male/Female	43 (44.8)/53 (55.2)
BMI	21.9 ± 3.9
Duration of disease (Months)	17.6 ± 16.0
Education (Years)	13.7 ± 2.9
Smoking status	
Current smokers	12 (12.5)
Past smokers	5 (5.2)
Never smokers	79 (82.3)
History of mental illness and other medical conditions	
Yes	25 (26.0)
No	71 (74.0)
Medication history	
Yes	59 (61.5)
No	37 (38.5)
Family history of mental illness	
Yes	18 (18.8)
No	78 (81.3)

FAS, full analysis set; BMI, Body mass index.

**Figure 1:**
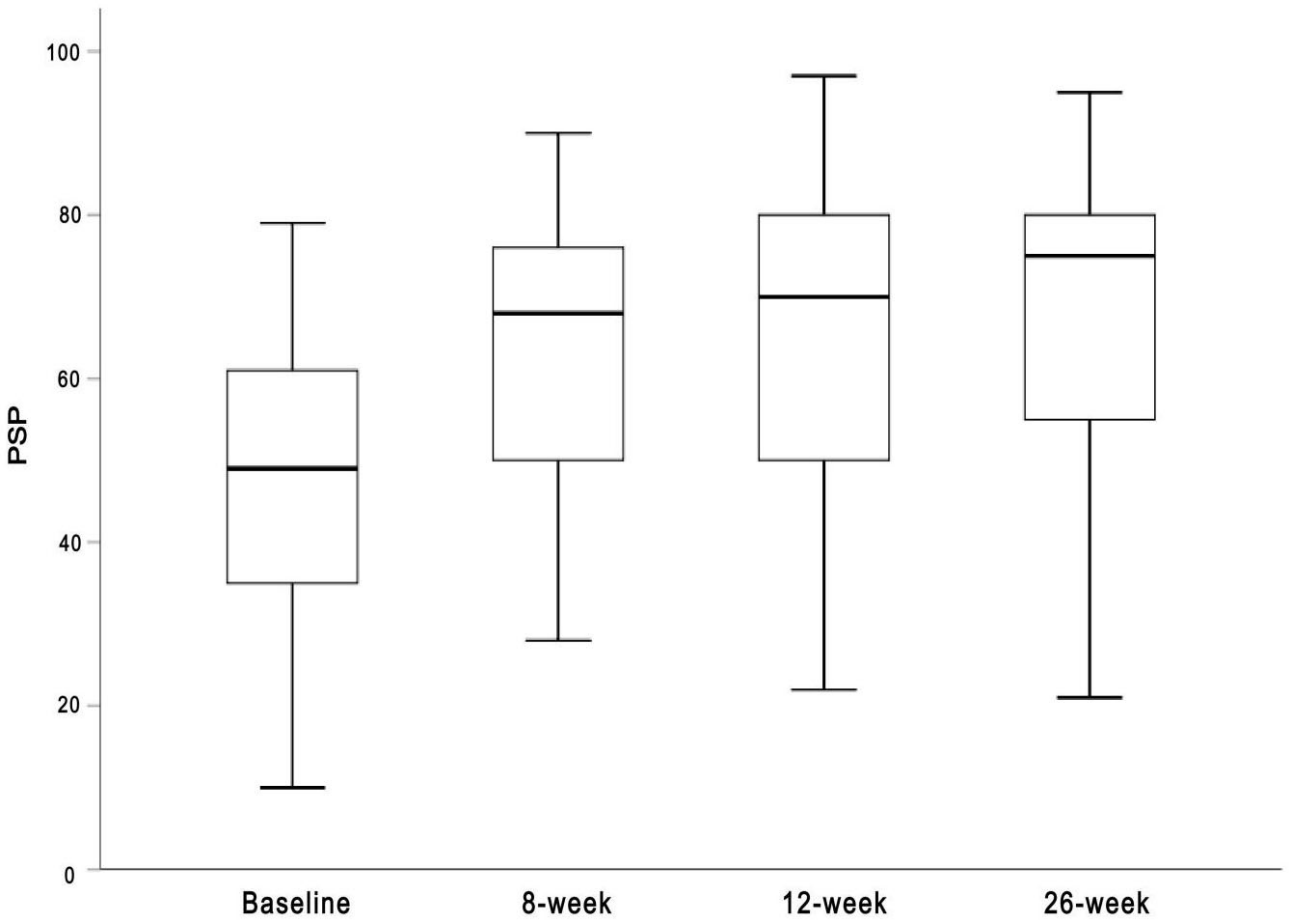
The trend of PSP total score during the 26-week follow-up period.

After treatment with blonanserin, all outcome measures demonstrated a significant improvement during the 26-week follow-up period. For PSP, 51 patients (53.1%, 51/96) completed the PSP scale measurements at baseline and week 26, and the total scores kept increasing from 46.6 ± 14.6 at the beginning of intervention to 69.4 ± 17.4 at week 26 (**Figure 1** and **Supplementary Table 2**). Among them, PSP total scores exceeding 70 were observed in 46.0% (23/50) at week 8 and 56.9% (29/51) at week 26.

At week 8, 38 patients showed the early response of PSP. To evaluate factors associated with the early response of PSP, we explored these associations using a multivariate logistic regression model (**Table 2**). Three factors, gender, PSP and MCCB GDS at baseline, had an impact on the early response of PSP, with odds ratios of 0.08 (95%CI: 0.01-0.98), 0.89 (95%CI: 0.80-0.99), and 0.38 (95%CI: 0.15-0.97), respectively, indicating that male patients with low PSP total score and low MCCB GDS at baseline were more likely to perform early response at week 8 (**Table 2**). Regarding the MCCB, its GDS result also indicates that patients exhibited an overall improvement in their neurocognitive impair status after the 26-week intervention (1.6 ± 1.1 at baseline, 1.3 ± 0.9 at week-26, p=0.007).

**Table 2:** Multifactorial analysis of factors associated with the early response of PSP score at 8-week.

Variables	OR	95%CI	p-value
Age (Year)	1.08	(0.94, 1.25)	0.26
Gender	0.08	(0.01, 0.98)	0.048
Course of disease (Month)	1.04	(0.97, 1.11)	0.30
Baseline PSP	0.89	(0.80, 0.99)	0.026
Baseline MCCB GDS	0.38	(0.15, 0.97)	0.04
Baseline PANSS	0.91	(0.82, 1.01)	0.07
Average daily dose of Blonanserin	0.96	(0.75, 1.23)	0.75

OR, odds ratio; CI, confidence interval; MCCB, MATRICS consensus cognitive battery; GDS, Global Deficit Score; PANSS, Positive and Negative Syndrome Scale; PSP, Personal and Social Performance.

PANSS total score significantly decreased from 85.8 ± 13.6 at baseline to 51.3 ± 15.5 at week 26; similarly, the PANSS positive, negative, and general psychopathology scores displayed the same decreasing trends (all p<0.001; **Table 3**). When compared to the PANSS total score at baseline, the reduction rates were 30.7% ± 27.2% at week 2 and consistently decreased to 61.7% ± 28.2% at week 26. A reduction rate of PANSS total scores exceeding 50% was observed in 58.8% (30/51) at week 8 and 72.5% (37/51) at week 26.

**Table 3:** PANSS scores during 26-week follow-up.

	PANSS-P	PANSS-N	PANSS-G	PANSS-score
Baseline	22.5 ± 5.6	20.6 ± 7.7	42.6 ± 7.9	85.8 ± 13.6
2-week	16.1 ± 5.7	17.7 ± 6.8	34.3 ± 8.6	68.0 ± 17.6
4-week	13.3 ± 5.5	17.2 ± 6.4	32.1 ± 9.2	62.6 ± 17.9
8-week	10.8 ± 4.0	16.1 ± 6.0	28.9 ± 8.1	55.0 ± 14.3
12-week	9.9 ± 4.1	15.4 ± 6.6	27.6 ± 8.6	52.9 ± 16.0
26-week	9.6 ± 4.0	14.9 ± 6.2	26.8 ± 8.1	51.3 ± 15.5
p^a^	<0.001	<0.001	<0.001	<0.001

^a:^ Repeated measure analysis of variance; PANSS-P, PANSS positive symptom; PANSS-N, PANSS negative symptom; PANSS-G, PANSS general psychopathology symptom.

At week 2, 50 patients demonstrated an improvement of more than 20% in the severity of schizophrenia. To evaluate factors associated with the early response of PANSS, we explored these associations using a multivariate logistic regression model (**Supplementary Table 3**). The early response of PANSS at week 2 was significantly influenced by the baseline PSP total score, with odds ratios of 1.05 (95%CI: 1.01, 1.10), suggesting that patients with a higher PSP total score at baseline had a greater likelihood of achieving early response at week 2. Additionally, the duration of disease (p = 0.07) and baseline MCCB GDS (p = 0.06) also exhibited a trend in impacting the early response of PANSS at week 2, with odds ratios of 0.96 (95%CI: 0.93, 1.00) and 0.59 (0.33, 1.01), respectively, indicating that patients with a shorter duration of disease and lower MCCB GDS at baseline may be more likely to exhibit early response. However, these impacts were not observed at week 26 (**Supplementary Table 3**).

**Figure 2:**
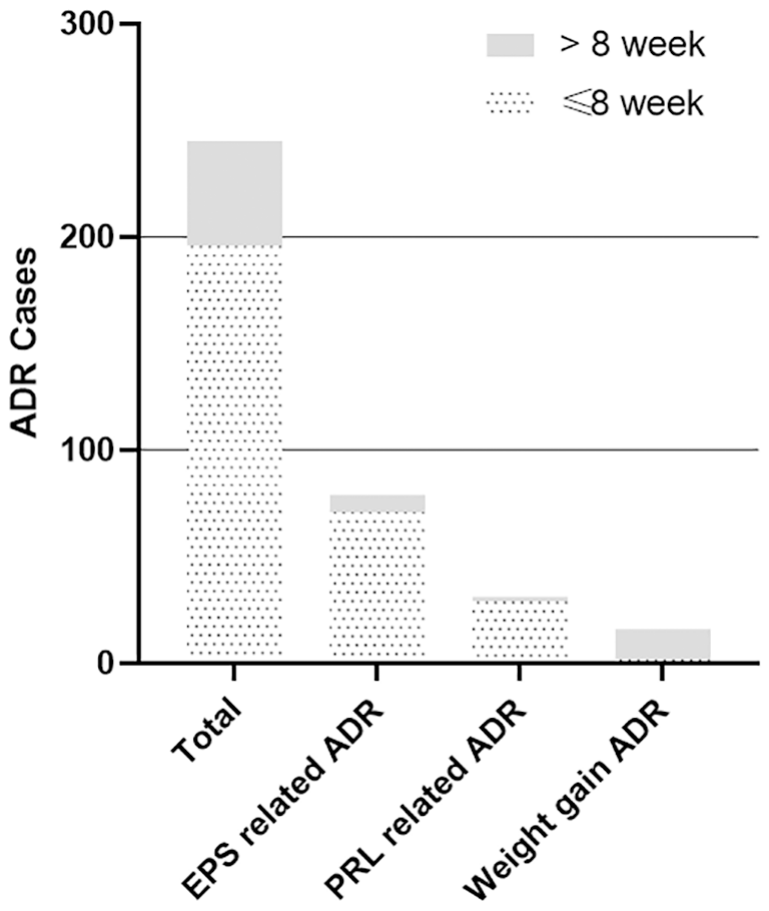
Incidence of ADRs during the 26-week follow-up period.

The adverse drug reactions (ADRs) were observed in 83.2% of patients (79/95), resulting in a total of 245 ADRs. Conversely, 16 patients (16.8%) did not report any ADRs. The major adverse effects observed in this clinical trial are presented in **Table 4**. Generally, the majority of ADRs were mild (40.0%, 38/95) or moderate (38.9%, 37/95). Only 4 patients reported severe ADRs (4.2%, 4/95). The most frequently reported ADRs included akathisia (33.7%), extrapyramidal symptoms (24.2%), and weight gain (16.8%). It is noteworthy that ADRs associated with extrapyramidal symptoms and elevated prolactin have significantly decreased after an 8-week follow-up. However, weight gain was more often observed after week 8, compared with that before week 8 (**Figure 2**).

**Table 4:** The adverse drug reactions (ADRs) at 26-week follow-up period (SS = 95).

ADRs	Number of patients (%)
Nervous system disorders	
Akathisia	32 (33.7%)
Extrapyramidal diseases	23 (24.2%)
Investigations	
Weight gain	16 (16.8%)
Elevated blood prolactin level	15 (15.8%)
Psychiatric disorders	
Anxiety	5 (5.3%)
Insomnia	5 (5.3%)
Cardiac disorders	
Palpitation	8 (8.4%)
Sinus bradycardia	5 (5.3%)
Gastrointestinal disorders	
Constipation	6 (6.3%)
Endocrine disorders	
Hyperprolactinemia	11 (11.6%)
General disorders and administration site conditions	
Fatigue	6 (6.3%)
Metabolic and nutritional diseases	5 (5.3%)

## 4 Discussion

In this prospective study of 96 participants from the Chinese population, the result shows that blonanserin significantly improved outcome measurements of PSP, MCCB and PANSS, in patients with first-episode schizophrenia during the 26-week follow-up period. Multivariate analyses further revealed that the factors such as age and gender have an impact on the early response, with PSP score at week 8 and PANSS total score at week 2, respectively. Moreover, the safety assessment indicated that treatment with blonanserin was primarily associated with mild adverse reactions. These findings suggest that blonanserin treatment demonstrates positive effects on social impairment, highlighting its potential as a promising treatment option for patients with first-episode schizophrenia.

The antipsychotic effects of blonanserin have been validated, and its introduction to the Chinese market reflects the recognition of its therapeutic efficacy (7, 17). However, there is limited research on its effects in terms of social functioning and neurocognition. In comparison to previous studies evaluating the effects of other second-generation antipsychotics (SGAs), blonanserin treatment in this clinical trial demonstrated a noteworthy improvement on social functioning during 26-week follow-up period, with a mean increment of 68.1% ± 103.7% (n=51). Nevertheless, the improvements observed in the PSP scores imply that blonanserin might exhibit a comparable efficacy in enhancing social and occupational functioning at week 8 when compared to other SGAs like risperidone, olanzapine, and aripiprazole (20). Furthermore, this study showed that positive effects on social functioning were evident as early as week 8, indicating a rapid response to blonanserin treatment (**Supplementary Table 2**). Although the enhancement in neurocognitive functioning was observable, it seemed to be relatively less prominent in contrast to the substantial effectiveness exhibited in social functioning.

Blonanserin holds significant promise as a therapeutic option, particularly in addressing social impairments in schizophrenia. However, while it showed efficacy in improving neurocognitive symptoms, further investigations and direct comparisons with other SGAs are necessary to fully ascertain its complete neurocognitive benefits. This expanded understanding of blonanserin’s effects on both social and neurocognitive functioning opens new avenues for tailoring treatment approaches and addressing the diverse needs of individuals with first-episode schizophrenia.

Regarding symptom severity, blonanserin showed substantial effectiveness in reducing symptom severity. The PANSS scores exhibited consistent decreasing trends over the 26-week follow-up period. Impressively, an early response to blonanserin treatment was observed as early as week 2, indicating its rapid action in alleviating schizophrenia symptoms. The reduction rates of PANSS scores were comparable to those reported for other SGAs (i.e., risperidone, haloperidol) in previous research (21–23). This highlights blonanserin’s potential as an effective treatment option for managing a wide range of schizophrenia symptoms.

This study also shows that, blonanserin generally has mild or moderate side effects such as akathisia, extrapyramidal diseases, and weight gain. Most ADRs occurred during the acute phase. However, after 8 weeks of blonanserin treatment, the incidence of major ADRs such as extrapyramidal symptoms (EPS) and elevated prolactin levels significantly decreased, with the exception of ADRs associated with weight gain (**Figure 2**). Comparisons of adverse effects between blonanserin and other SGAs in clinical trials have shown differences in the incidence and severity of specific side effects (4, 20, 24–26). For example, blonanserin exhibited a reduced risk of prolactin elevation and orthostatic hypotension compared to risperidone. Specifically, a network meta-analysis showed that blonanserin was the first-ranked antipsychotic for the lowest risk of weight change (27). However, blonanserin was associated with a higher occurrence of akathisia when contrasted with risperidone (26), which could be attributed to its high affinity for dopamine D2 receptors (7). ADRs should be carefully considered when selecting an antipsychotic, the patient’s individual profile and preferences also should be taken into account.

The current study has several strengths. A notable strength of this study was its prospective, multi-centre, single-arm design, which allowed for the assessment of blonanserin’s efficacy in a relatively large sample of patients with first-episode schizophrenia (n=96). The 26-week follow-up period provided valuable insights into the shorter term (i.e., 2-8 weeks) and longer-term effects of blonanserin treatment on various outcome measures. Additionally, the use of standardized assessment tools, such as the PSP, MCCB, and PANSS, enhances the reliability and validity of the findings.

However, some limitations should be acknowledged. Firstly, the sample size for this study did not reach the original target. The planned sample size in the protocol was calculated based on previously reported PSP changes. However, the observed PSP improvement in the current trial was higher than the estimated value, which could potentially mitigate the impact of insufficient recruitment. Secondly, most subjects in this trial were enrolled during the COVID pandemic, and due to the epidemic prevention and control requirements, there was a relatively large loss of follow-up in this trial, introducing potential bias, such as reduced statistical power or increased risk of over optimistic estimate of efficacy and safety, which may have led the bias in outcome assessment. Lastly, current analysis mainly focused on social impairment, but the findings also suggest the effects of blonanserin on cognitive deficits, although the changes in MCCB GDS may not comprehensively reflect improvements in neurocognition. Our team plans to prepare another paper specifically examining the neurocognitive improvements induced by blonanserin across each domain in the MCCB.

The positive outcomes observed in this study suggest that blonanserin could be a valuable treatment option for patients with first-episode schizophrenia. Its efficacy in improving social functioning, as well as neurocognitive and symptom severity, may contribute to enhanced overall functional outcomes and quality of life for patients in the early stages of the illness. The observed social benefits of blonanserin may have particular relevance, as social impairment is a significant challenge in schizophrenia. These results provide clinicians with additional evidence supporting the use of blonanserin as a viable treatment option for patients with first-episode schizophrenia, particularly in terms of improving functioning and symptoms for early response, enhancing social functioning and alleviating symptom severity.

In conclusion, this clinical trial provides evidence for the positive effects of blonanserin treatment on social functioning in patients with first-episode schizophrenia. However, further large-scale randomized controlled trials are required to validate these results and compare the efficacy and safety of blonanserin with other antipsychotics. Nevertheless, the current findings support the clinical use of blonanserin as a potentially valuable treatment option for individuals with first-episode schizophrenia, with the potential to improve functional outcomes and ameliorate symptoms.

## Data availability statement

The raw data supporting the conclusions of this article will be made available by the authors, without undue reservation.

## Ethics statement

The studies involving humans were approved by Peking University Sixth Hospital. The studies were conducted in accordance with the local legislation and institutional requirements. The participants provided their written informed consent to participate in this study.

## Author contributions

TG: Formal analysis, Investigation, Writing – original draft. HD: Formal analysis, Investigation, Writing – review & editing. JS: Formal analysis, Investigation, Writing – review & editing. BW: Formal analysis, Investigation, Writing – review & editing. ZL: Formal analysis, Investigation, Writing – review & editing. FY: Formal analysis, Investigation, Writing – review & editing. LW: Formal analysis, Investigation, Writing – review & editing. SH: Formal analysis, Investigation, Writing – review & editing. XW: Formal analysis, Investigation, Writing – review & editing. HL: Conceptualization, Methodology, Formal analysis, Writing – review & editing. CP: Formal analysis, Investigation, Writing – original draft. XY: Conceptualization, Methodology, Formal analysis, Investigation, Supervision, Writing – original draft.
