# Supplementary material for: Improvement of social functioning in patients with first-episode schizophrenia using blonanserin treatment: a prospective, multi-centre, single-arm clinical trial
Source: Front Psychiatry. 2024 Mar 20;15:1345978. doi: 10.3389/fpsyt.2024.1345978 (PMC10988613; doi:10.3389/fpsyt.2024.1345978)
Supplement: Supplementary file 1 [file Table_1.docx]

# 1 Supplementary Tables

**Supplementary Table 1. The time points at which the assessments were conducted**

|  | **Screening** | **0-week** | **2-week** | **4-week** | **8-week** | **12-week** | **26-week** |
| --- | --- | --- | --- | --- | --- | --- | --- |
| **PSP** |  | **×** |  |  | **×** | **×** | **×** |
| **MCCB** |  | **×** |  |  | **×** |  | **×** |
| **PANSS** | **×** |  | **×** | **×** | **×** | **×** | **×** |

PSP: Personal and Social Performance; MCCB: MATRICS consensus cognitive battery; PANSS: Positive and Negative Syndrome Scale

**Supplementary Table 2. The PSP total score and its change at 8, 12 and 26 weeks**

|  | **PSP total score (Mean±SD)** | **Change of PSP total score** | **p-value^a^** | **p-value^b^** |
| --- | --- | --- | --- | --- |
| Baseline | 46.6±14.6 | - | - | < 0.001 |
| 8-week | 63.3±15.4 | 15.6±15.9 | < 0.001 |  |
| 12-week | 66.7±17.5 | 18.6±19.3 | < 0.001 |  |
| 26-week | 69.4±17.4 | 22.0±19.9 | < 0.001 |  |

a: Wilcoxon rank sum test; b: repeated measures analysis of variance; PSP: Personal and Social Performance; SD: standard deviation

| **Supplementary Table 3. Multivariate analysis of factors associated with the early response of PANSS at 2 and response of PANSS at 26 weeks** | | | | | | | |
| --- | --- | --- | --- | --- | --- | --- | --- |
|  | **2-week** | | |  | **26-week** | | |
| **Variables** | **OR** | **95%CI** | **p-value** |  | **OR** | **95%CI** | **p-value** |
| Age (Year) | 1.04 | (0.96, 1.12) | 0.39 |  | 1.02 | (0.95, 1.10) | 0.53 |
| Gender | 0.57 | (0.18, 1.68) | 0.32 |  | 0.81 | (0.30, 2.14) | 0.67 |
| Duration of disease (Month) | 0.96 | (0.93, 1.00) | 0.07 |  | 0.99 | (0.96, 1.03) | 0.59 |
| Baseline PSP | 1.05 | (1.01, 1.10) | 0.01 |  | 1.02 | (0.99, 1.06) | 0.20 |
| Baseline MCCB GDS | 0.59 | (0.33, 1.01) | 0.06 |  | 0.74 | (0.43, 1.24) | 0.25 |
| Baseline PANSS | 1.02 | (0.97, 1.07) | 0.45 |  | 1.04 | (1.00, 1.09) | 0.08 |
| Mean daily dose of Blonanserin in the first 2 weeks | 1.01 | (0.91, 1.14) | 0.81 |  | 0.95 | (0.85, 1.07) | 0.43 |
| OR: odds ratio; CI: confidence interval; MCCB: MATRICS consensus cognitive battery; GDS: Global Deficit Score; PANSS: Positive and Negative Syndrome Scale; PSP: Personal and Social Performance | | | | | | | |
